# Black Digits Matter: A Multispecialty Enigma

**DOI:** 10.7759/cureus.55133

**Published:** 2024-02-28

**Authors:** Abhijeet Agrawal, Jahnabi Bhagawati, Sunil Kumar, Sourya Acharya

**Affiliations:** 1 Department of Medicine, Jawaharlal Nehru Medical College, Wardha, IND

**Keywords:** antiphospholipid antibody, scleroderma, systemic lupus erythematosus, connective tissue disease, autoimmune, digital ulcer, raynaud's phenomenon, digital ischemia

## Abstract

Introduction

Digital ischemia is alike any other visceral ischemic event leading to severe tissue damage ultimately causing necrosis of the involved extremity. It’s like a preview of the upcoming systemic disorder and can present itself in any specialty and hence everyone, be it a physician or a surgeon must be primed toward how to proceed with a case of digital ischemia. In this case series, we present six such cases that presented with digital ischemic events either as a sole presentation or were followed by other systemic manifestations that led to their evaluation and ultimately the etiology behind it.

Material and method

Patients visiting Rheumatology OPD with complaints suggestive of digital ischemia were included in this study. All patients underwent thorough history taking and clinical examination to establish the cause of digital ischemia. Patients with probable infective, trauma, cardiac, and drug-induced causes and malignancies were excluded. As per probable autoimmune causes, patients underwent evaluation via antinuclear antibodies by immunofluorescence (ANA by IF), antiphospholipid antibodies like lupus anticoagulant (LAC), anticardiolipin antibodies (AcL) and anti Beta2GP1 antibodies, extractable nuclear antigens (ENA) and in cases of suspected vasculitis doppler ultrasound and angiography.

Results

Six patients were identified as cases primarily presenting with digital ischemia or with a prior history of digital ischemia. Two patients were of the pediatric age group, one 16-year-old male presenting with acute arthritis and a history of digital ischemia one year back, and the other was a 12-year-old female with blackening of the second toe in her left foot with a history of similar complaints in the left great toe for which she underwent amputation of that toe. Other four cases were of the adult age group, with two cases of scleroderma, one with systemic lupus erythematosus, and one with Takayasu arteritis. All of these patients primarily presented to departments other than rheumatology.

Conclusion

Digital ischemia is a pan-specialty problem with the etiologies spreading across a vast spectrum of rheumatological disorders, many of which may present to different specialties initially, later discovered to be part of the systemic manifestation of autoimmune diseases. Hence, it becomes imperative to have a rheumatological perspective in these cases of digital ischemia which all specialities should be aware of, and timely referral may prevent permanent loss of the digits and in some cases the entire limb.

## Introduction

Adequate blood flow is of utmost importance for the vitality of any living tissue. Loss of this homeostasis leads to extreme agony and can be disfiguring. It also leads to decreased quality of life and may prove fatal for the involved tissue. Digital ischemia is like any cardiovascular or pulmonary ischemic event and should be dealt with extreme caution in order to prevent any complications. Such incidences of digital ischemia may be seen across a wide spectrum of rheumatological disorders and hence it becomes imperative to have a thorough concept of possible causes of digital ischemia [1]. Connective tissue diseases are characterized by tissue inflammation including inflammation of the vessel walls which may alter the homeostasis thereby making the vascular endothelium pro-thrombotic [2,3].

Connective tissue diseases like systemic lupus erythematosus (SLE), antiphospholipid antibody syndrome (APLA/APS), systemic sclerosis (SSc), and even systemic vasculitis may present with critical digital ischemia. About 9% of SSc and 5% of SLE patients present with digital gangrene [4]. APLA presents as pregnancy morbidity and/or thrombotic events due to the presence of antibodies like lupus anticoagulant, anti-cardiolipin antibodies, and anti-Beta2GP1 antibodies. They may present as both venous and arterial thrombosis, venous being the commonest mode of presentation, especially in the pediatric population [5]. Thirty-two percent of children may present with arterial thrombosis. 6% may have small vessel involvement leading to digital ischemia or thrombotic microangiopathy. They may also present with non-thrombotic neurological events. Pediatric APLA is usually reserved for patients under 18 years of age [6]. SLE usually presents with cutaneous and musculoskeletal manifestations but may affect any and every organ [7]. Among cutaneous manifestations, apart from classical acute, subacute, and chronic cutaneous lupus manifestations, 1.3% of patients may develop digital ischemia. These can ultimately lead to loss of the affected digit due to necrosis. The pathologic mechanism behind this dreaded complication can be accelerated atherosclerosis, vasculitis, thromboembolic events, or vasospasm (Raynaud’s phenomenon) [8,9]. Raynaud’s phenomenon is often triggered due to cold weather, infection, drugs like sympathomimetics, and connective tissue diseases [10]. Previous data has shown very few patients develop digital gangrene due to SLE. Adding to that, it’s still rarer for it to be the sole presenting feature of the disease, seen only in 0.2%. Few reports revealed that on average the period between the onset of SLE to digital gangrene varies widely between 1 and 18 years [8,9,11]. Systemic sclerosis is another connective tissue disease characterized by Raynaud's phenomenon which leads to vasospasm and may be the presenting feature in the majority of patients. This may lead to digital ulcerations at the fingertips. The pathogenic mechanism behind this stems from vessel wall proliferation and thrombosis [12]. Takayasu arteritis represents vasculitis involving the large vessels, usually the aorta and branches. Digital ischemia as a presentation of Takayasu is very rare but may be a consequence of embolism or vasculitis of smaller vessels [13].

Here we present a series of cases that came to Rheumatology OPD via referral from various branches of medicine and surgery, all with digital ischemia as their predominant manifestations.

## Materials and methods

Patients who came to Rheumatology OPD (Jawaharlal Nehru Medical College, Wardha) between August 2023 and December 2023 with complaints suggestive of digital ischemia were included in this study. All patients underwent thorough history taking and clinical examination to establish the cause of digital ischemia. Patients with probable infective, trauma, cardiac, and drug-induced causes and malignancies were excluded. As per the probable autoimmune cause, patients were investigated for the presence of antinuclear antibodies by immunofluorescence (ANA by IF), antiphospholipid antibodies like lupus anticoagulant (LAC), anticardiolipin antibodies (AcL), and anti-Beta2GP1 antibodies, extractable nuclear antigens (ENA) and in cases of suspected vasculitis Doppler ultrasound and angiography. 

## Results

Case 1

**Figure 1:**
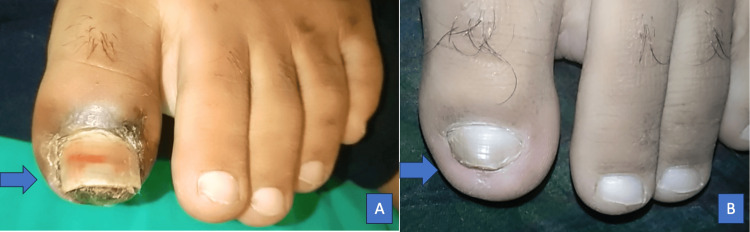
(A) Left Great Toe Showing Ischemic Discoloration. (B) Post-intravenous Thrombolysis Image

**Figure 2:**
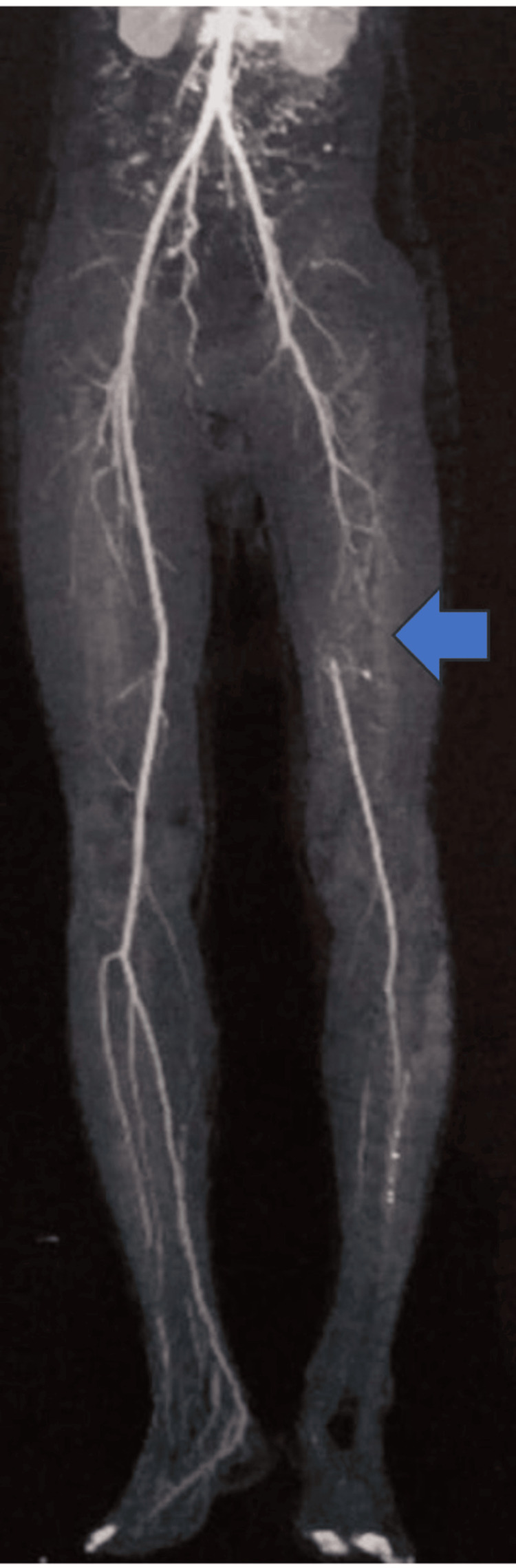
CT Angiogram Showing Long Segment Obstruction in the Left Superficial Femoral Artery with Collateral Formation (Arrow Showing No Contrast between Proximal and Distal Superficial Femoral Artery) CT: Computerized Tomography

A 16-year-old boy came with complaints of pain and swelling in multiple joints for two months associated with morning stiffness. He also had a history of mild-grade fever on and off, with no other symptoms suggestive of any infection. Arthritis involves both large and small joints of all four limbs in a symmetrical fashion. There was no history of back pain, eczema, psoriasis, or any features suggestive of inflammatory bowel disease, weight loss, purpura, oral ulcers, Raynaud's phenomenon, or serositis. On further history taking, the boy’s father revealed blackening of the great toe in his left foot in the previous year (Figure 1A). It was sudden in onset and gradually progressive associated with pain but no fever. There was no history of trauma, purulent pus discharge, bleeding, or loss of sensation around the affected area. The dermatologist ruled out any local site infection. The patient was then referred to intervention radiology by their primary physician. On investigation, the angiography revealed occlusion of the lumen of the left superficial femoral artery (Figure 2). The boy underwent intraarterial thrombolysis and within a few weeks, the discoloration disappeared completely (Figure 1B). The patient was kept on dual antiplatelet (Tablet Ecosprin 75 mg once daily and Tablet Clopidogrel 150 mg once daily) since then. With this added history of arterial thrombus, the patient was evaluated and found to be dual positive for antiphospholipid antibodies (lupus anticoagulant and anti-cardiolipin IgM), ANA by IF was positive and ENA profile showed no other associated antibodies (Table 1). The patient was started on the Tablet warfarin 3 mg once daily which was increased to 3 mg and 4 mg once every alternate day to keep the INR (International Normalized Ratio) between 2 and 3, after an overlap with low-molecular-weight heparin 40 mg s.c. once daily and Tablet methotrexate 15 mg once weekly on Sunday with folic acid 5 mg once every day except Sunday for arthritis. At present, the patient’s arthritis has resolved and has had no new thrombotic events. 

**Table 1:** Investigations (Case 1) Hb: Hemoglobin; TLC: Total Leucocyte Count; Cr: Serum Creatinine; SGOT: Serum Glutamic Oxaloacetic Transaminase; SGPT: Serum Glutamic Pyruvic Transaminase; APTT: Activated Partial Thromboplastin Time; RBC: Red Blood Cells; ESR: Erythrocyte Sedimentation Rate; CRP: C-Reactive Protein; ANA by IF: Anti-nuclear Antibody by Immunofluorescence; 1:320: indicates the titre of ANA by IF; 1+: Indicates the strength of immunofluorescence; speckled: indicates the pattern of immunofluoroscence; ENA: Extractable Nuclear Antigen; LAC: Lupus Anticoagulant; Beta 2 GP1: Beta 2 Glycoprotein

Investigations	Observed Values	Reference Values
Hb (g/dl)	12.1	13 to 17 (male), 12 to15 (female)
TLC (per microL)	4500	4000 to 10000
Platelets (per microL)	2.34 lac	1.5 to 4 lac
Cr (mg/dl)	0.5	0.6 to 1.2 (male), 0.5 to 1.0 (female)
SGOT (U/l)	22	17 to 59 (male), 14 to 36 (female)
SGPT (U/l)	24	< 50 (male), < 35 (female)
Globulin (g/dl)	3.7	2.5 to 3.5
Direct Coombs test	Negative	Negative
APTT (seconds)	56	< 30 seconds
Urine protein	Nil	Nil
Urine RBC	Nil	Nil
ESR (mm/hr)	88	1 to 10 (male), 3 to 15 (female)
CRP (mg/dl)	12.9	0 to 5
ANA By IF	1: 320	Negative
	1+	
	Speckled	
ENA profile	Negative	Negative
Anti-phospholipid antibodies	LAC Positive	Negative
	Anti Beta2 GP1 IgM = 78 gpl/mpl	Beta2GP1 IgM = < 40

Case 2

**Figure 3:**
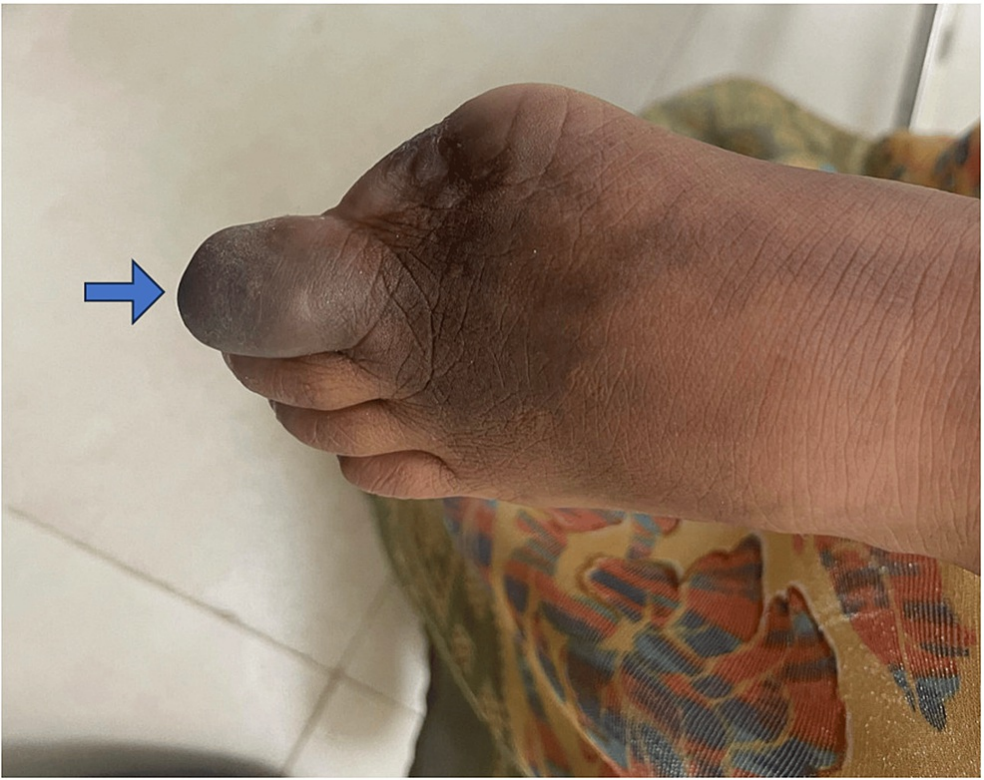
Left Second Toe Showing Ischemic Changes

A 12-year-old female child was brought to the OPD with complaints of blackish discoloration of the left second toe for two weeks (Figure 3). There was no history of fever, pain in the toe, discharge, or trauma. The patient had similar complaints three months back in her left great toe which rapidly progressed to gangrene, and the patient had to undergo amputation of the great toe for the same. There was no history of any recurrent fever, photosensitive rash, Raynaud's phenomenon, oral ulcers, serositis, neurological event, or genital ulcers. The patient underwent an ultrasound Doppler which showed normal blood flow in the foot. An MRI foot was done which showed no abnormality. To rule out any autoimmune etiology ANA by IF was done which came to be positive, and the ENA profile showed no associated antibodies. Her Anticardiolipin Antibody (AcL) IgM type was positive (Table 2). Based on these investigations, a diagnosis of antiphospholipid antibody syndrome was made and the patient was started on low-molecular-weight heparin 40 mg subcutaneously once daily for five days overlapped with Tablet Warfarin 2 mg once daily from 3rd day, keeping the INR between 2 and 3 and Tablet Hydroxychloroquine 200 mg twice daily. At present after three months, the patient is clinically stable with no new lesions. 

**Table 2:** Investigations (Case 2) Hb: Hemoglobin; TLC: Total Leucocyte Count; Cr: Serum Creatinine; SGOT: Serum Glutamic Oxaloacetic transaminase; SGPT: Serum Glutamic Pyruvic Transaminase; APTT: Activated Partial Thromboplastin Time; RBC: Red Blood Cells; ESR: Erythrocyte Sedimentation Rate; CRP: C-Reactive Protein; ANA by IF: Anti-Nuclear Antibody by Immunofluorescence; 1:160: indicates the titer of ANA by IF; 1+: indicates the strength of immunofluorescence; speckled: indicates the pattern of immunofluorescence; ENA: Extractable Nuclear Antigen; AcL: Anticardiolipin Antibody

Investigations	Observed Values	Reference Values
Hb (g/dl)	11.2	13 – 17 (male), 12 – 15 (female)
TLC (per microL)	5300	4000 – 10000
Platelets (per microL)	3.2 lac	1.5 – 4 lac
Cr (mg/dl)	0.6	0.6 – 1.2 (male), 0.5 – 1.0 (female)
SGOT (U/l)	30	17 – 59 (male), 14 – 36 (female)
SGPT (U/l)	21	Less than 50 (male), Less than 35 (female)
Globulin (g/dl)	3.2	2.5 – 3.5
Direct Coombs test	Negative	Negative
APTT (seconds)	60	Less than 30 seconds
Urine protein	Nil	Nil
Urine RBC	Nil	Nil
ESR (mm/hr)	76	1 – 10 (male), 3 – 15 (female)
CRP (mg/dl)	9.8	0 – 5
ANA By IF	1: 160	Negative
	1+	
	Speckled	
ENA profile	Negative	Negative
Antiphospholipid Antibody, AcL IgM (gpl/mpl)	62	Less than 40

Case 3

**Figure 4:**
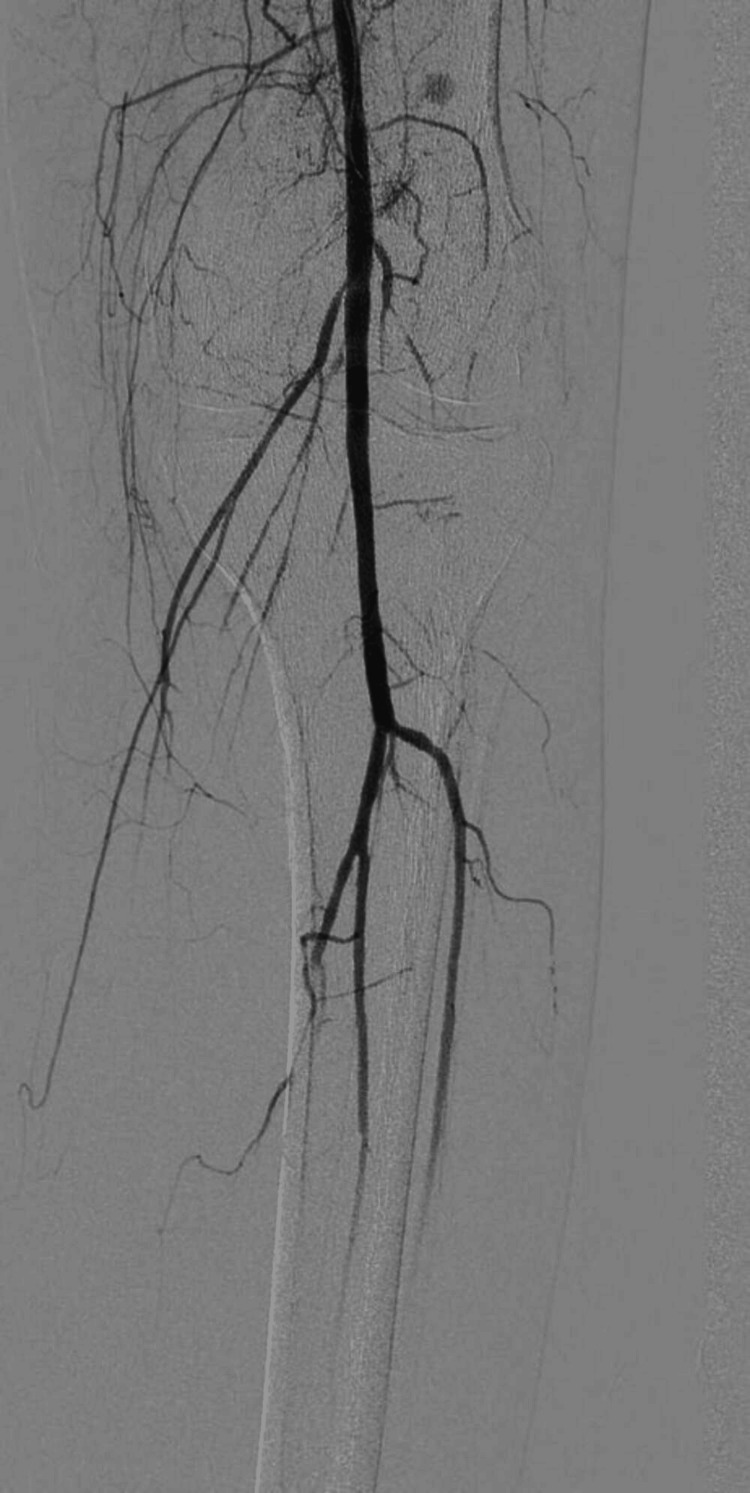
Angiography Showing Blood Flow in the Lower Limb Above the Knee

**Figure 5:**
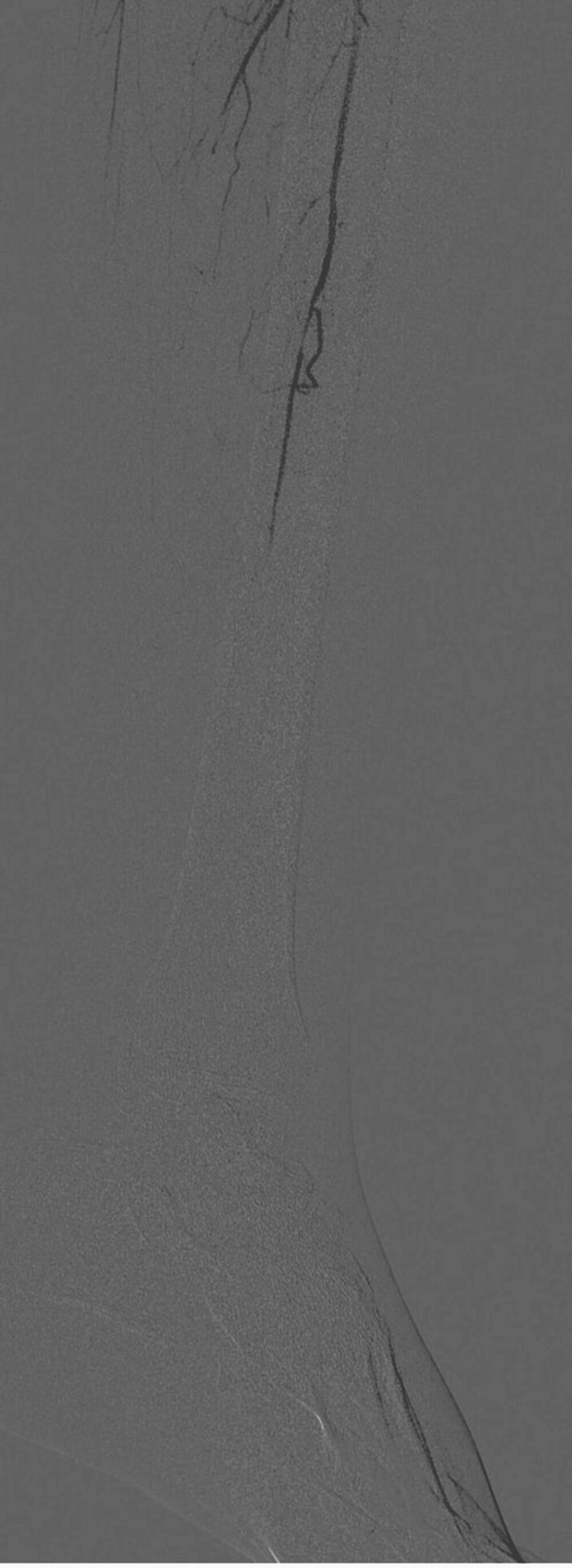
Angiogram Showing No Blood Flow in the Right Leg Below the Knee

**Figure 6:**
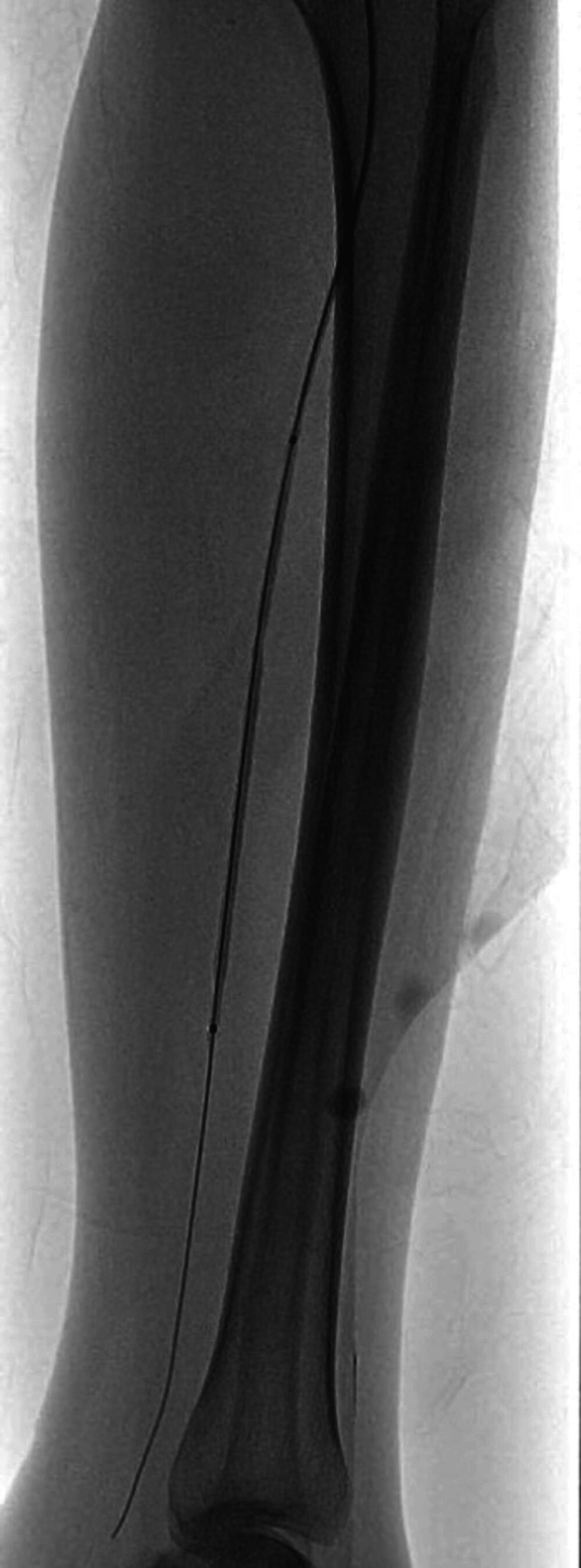
Post-balloon Angioplasty Restoration of Blood Flow in the Right Leg Below the Knee

A 26-year-old female was referred to Rheumatology for complaints of arthritis from surgery ICU. The patient had recently undergone below-knee amputation of the left leg following gangrenous changes which started one month back. The patient was previously diagnosed as a case of Rheumatoid arthritis six years back and was treated with methotrexate and folic acid. During the post-operative recovery period, the patient had a flare of arthritis. The patient was thoroughly examined, she had multiple tender and swollen joints. On examination of peripheral pulses, she had a feeble pulse in the right Dorsal Pedis artery and bilateral brachial artery. The examination also revealed the presence of bruit in bilateral femoral arteries. Ultrasound Doppler showed reduced flow in the left brachial artery and left radial artery. CT angiography was done which showed normal blood flow above the knee (Figure 4) and narrowing of the lumen of the vessels of the right leg below the knee (Figure 5), which was corrected later via balloon angioplasty (Figure 6). The patient had an ESR of 110 (Table 3) and a fever on and off. Later during the hospital stay, the patient also developed claudication of the left arm and headaches. The patient was treated for Takayasu arteritis with intravenous pulse glucocorticoids (Injection of Methylprednisolone 500 mg intravenous once daily for three days). Post-pulse therapy of steroids, the patient was kept on tapering dosage of oral steroids (Tablet Prednisolone at 1 mg/kg/day) along with Tab Methotrexate 15 mg once every Saturday and Tablet Folic Acid 5 mg once every day except Saturday. One month post-discharge, her musculoskeletal symptoms resolved and her ESR also came down to 33. 

**Table 3:** Investigations (Case 3) Hb: Hemoglobin; TLC: Total Leucocyte Count; Cr: Serum Creatinine; SGOT: Serum Glutamic Oxaloacetic transaminase; SGPT: Serum Glutamic Pyruvic Transaminase; RBC: Red Blood Cells; ESR: Erythrocyte Sedimentation Rate; CRP: C-Reactive Protein

Investigations	Observed Values	Reference Values
Hb (g/dl)	10.2	13 – 17 (male), 12 – 15 (female)
TLC (per microL)	17600	4000 – 10000
Platelets (per microL)	3.89 lac	1.5 – 4 lac
Cr (mg/dl)	0.9	0.6 – 1.2 (male), 0.5 – 1.0 (female)
SGOT (U/l)	44	17 – 59 (male), 14 – 36 (female)
SGPT (U/l)	38	< 50 (male), < 35 (female)
Globulin (g/dl)	4.1	2.5 – 3.5
Urine protein	Nil	Nil
Urine RBC	Nil	Nil
ESR (mm/hr)	110	1 – 10 (male), 3 – 15 (female)
CRP (mg/dl)	14.9	0 – 5

Case 4

**Figure 7:**
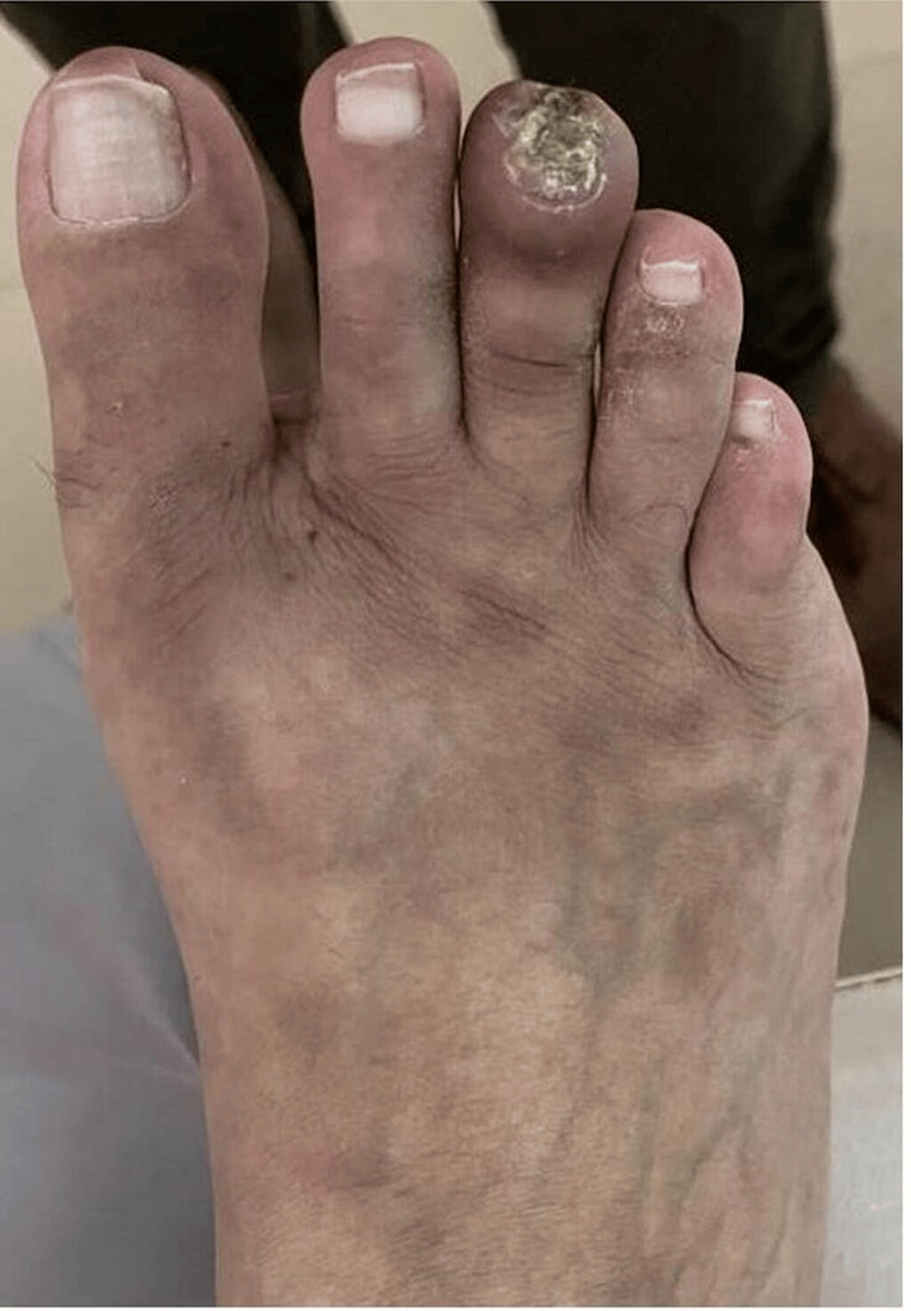
Right Third Toe Showing Ischemic Changes

**Figure 8:**
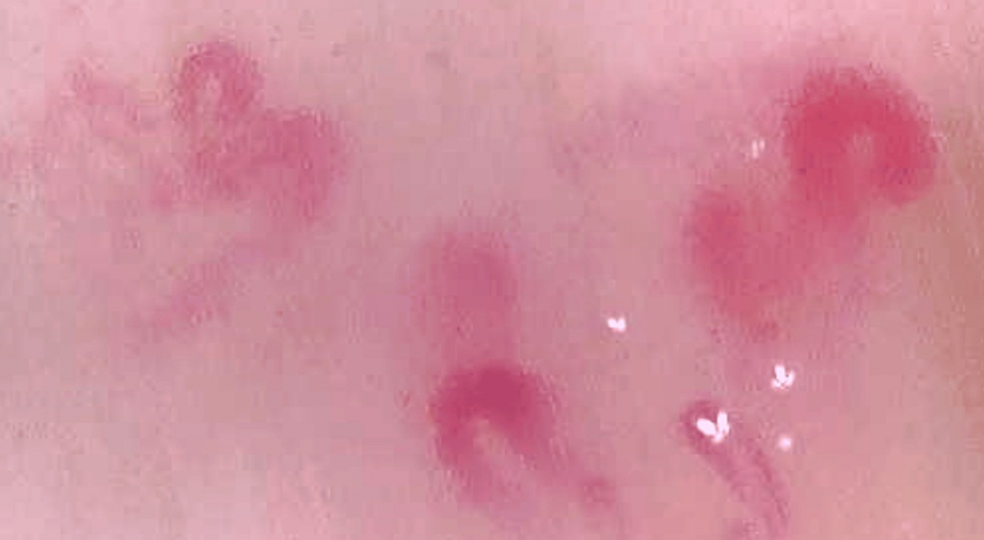
Nail Fold Capillaroscopy Showing Giant Capillaries

A 30-year-old female came with complaints of swelling in both hands-on and off for two months. The swelling was diffused involving the hand from the wrist to the fingers. On history, she revealed joint pains early morning involving predominantly the upper limb joints both small and large in a symmetrical fashion associated with morning stiffness for two years. Multiple fingers showed digital pits and scars. The right third toe had dried up old gangrenous changes at the tip (Figure 7). Ultrasound Doppler showed normal blood flow to the digits. The forehead showed pepper pot skin and mouth opening was also restricted. The patient also had a history of difficulty in swallowing two years back for which a gastroenterologist evaluated her. Nail fold capillaroscopy showed few typical giant capillaries with areas of capillary dropouts suggesting a late scleroderma pattern (Figure 8). The modified Rodnan skin score was 19. On systemic examination, there were no crepitations on auscultation and no signs of pulmonary hypertension. Investigations revealed a positive ANA by IF, and ENA profile positive for anti-Scl-70 antibody (Table 4). The patient was started on treatment for diffuse scleroderma with vasodilators (Tablet Nifedipine retard 10 mg twice daily), antiplatelets (Tablet Ecosprin 75 mg once daily) and prokinetic agents (Tablet Pantoprazole 40 mg + Domperidone 30 mg once daily) and currently, her disease activity is under control with no new digital ischemic events or systemic flares. 

**Table 4:** Investigations (Case 4) Hb: Hemoglobin; TLC: Total Leucocyte Count; Cr: Serum Creatinine; SGOT: Serum Glutamic Oxaloacetic Transaminase; SGPT: Serum Glutamic Pyruvic Transaminase; RBC: Red Blood Cells; ESR: Erythrocyte Sedimentation Rate; CRP: C-Reactive Protein; ANA by IF: Anti-nuclear Antibody by Immunofluorescence; 1:320: indicates the titre of ANA by IF; 2+: indicates the strength of immunofluorescence; speckled: indicates the pattern of immunofluorescence; ENA: Extractable Nuclear Antigen; ScL-70: Scleroderma antibody/Anti Topoisomerase Antibody

Investigation	Observed Values	Reference Values
Hb (g/dl)	9.8	13 – 17 (male), 12 – 15 (female)
TLC (per microL)	8900	4000 – 10000
Platelets (per microL)	2.10 lac	1.5 – 4 lac
Cr (mg/dl)	0.5	0.6 – 1.2 (male), 0.5 – 1.0 (female)
SGOT (U/l)	54	17 – 59 (male), 14 – 36 (female)
SGPT (U/l)	32	< 50 (male), < 35 (female)
Globulin (g/dl)	3.9	2.5 – 3.5
Urine protein	Nil	Nil
Urine RBC	Nil	Nil
ESR (mm/hr)	70	1 – 10 (male), 3 – 15 (female)
CRP (mg/dl)	8.9	0 – 5
ANA By IF	1:320	Negative
	2+	
	Speckled	
ENA profile	Anti Scl-70 positive	Negative

Case 5

**Figure 9:**
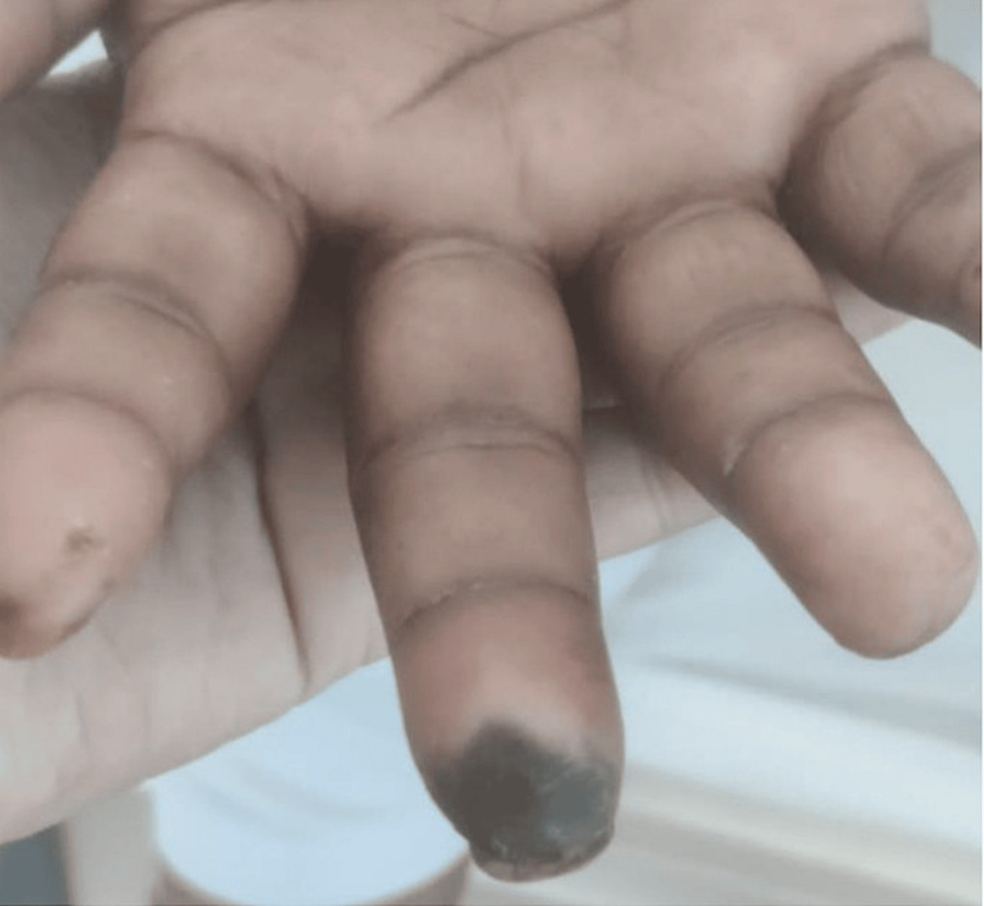
Right Middle Finger Distal Phalanx Showing Necrotic Changes

A 40-year-old female was referred from the Respiratory medicine department to Rheumatology for evaluation of a possible ILD (interstitial lung disease) with CTD (connective tissue disease). The patient had a history of dry cough for two years which was insidious in onset and gradually progressive and mild breathlessness on exertion (grade II mMRC). The patient also had a history of multiple joint pains with swelling and early morning stiffness for more than 30 minutes for six months. The left middle finger had started showing ischemic changes for two weeks (Figure 9). No history of Raynaud's phenomenon, oral ulcers, genital ulcers, muscle weakness, recurrent fever, or any other systemic features. On examination, there was no obvious muscle weakness, the facial rash had faded, and the patient had multiple tender and swollen joints. On investigations, ANA by IF was positive and ENA profile showed anti centromere antibody (Table 5). HRCT was done which showed findings of mild ILD with no new changes when compared to the previous HRCT done one year back. PFT (pulmonary function test) was done which showed mild restrictive changes. Echocardiography revealed mild pulmonary artery hypertension. A diagnosis of limited scleroderma was made and the patient was started on Tablet Mycophenolate Mofetyl 500 mg twice daily, Tablet Tadalafil 10 mg once daily, Tablet Ecosprin 75 mg once daily, and the patient was asked to keep her body and extremities warm, especially during winters. 

**Table 5:** Investigations (Case 5) Hb: Hemoglobin; TLC: Total Leucocyte Count; Cr: Serum Creatinine; SGOT = Serum Glutamic Oxaloacetic Transaminase; SGPT: Serum Glutamic Pyruvic Transaminase; RBC: Red Blood Cells: ESR: Erythrocyte Sedimentation Rate; CRP: C-Reactive Protein; ANA by IF: Anti-nuclear Antibody by Immunofluorescence; 1:80: indicates the titre of ANA by IF; 1+: indicates the strength of immunofluorescence; Centromere: indicates the pattern of immunofluorescence; ENA: Extractable Nuclear Antigen; CENP-B: Centromere Protein B

Investigations	Observed Values	Reference Value
Hb (g/dl)	9.0	13 – 17 (male), 12 – 15 (female)
TLC (per microL)	7600	4000 – 10000
Platelets (per microL)	2.12 lac	1.5 – 4 lac
Cr (mg/dl)	1.1	0.6 – 1.2 (male), 0.5 – 1.0 (female)
SGOT (U/l)	34	17 – 59 (male), 14 – 36 (female)
SGPT (U/l)	31	< 50 (male), < 35 (female)
Globulin (g/dl)	3.7	2.5 – 3.5
Urine protein	Nil	Nil
Urine RBC	Nil	Nil
ESR (mm/hr)	56	1 – 10 (male), 3 – 15 (female)
CRP (mg/dl)	6.9	0 – 5
ANA By IF	1:80	Negative
	1+	
	Centromere	
ENA profile	Anti CENP-B Positive	Negative

Case 6

**Figure 10:**
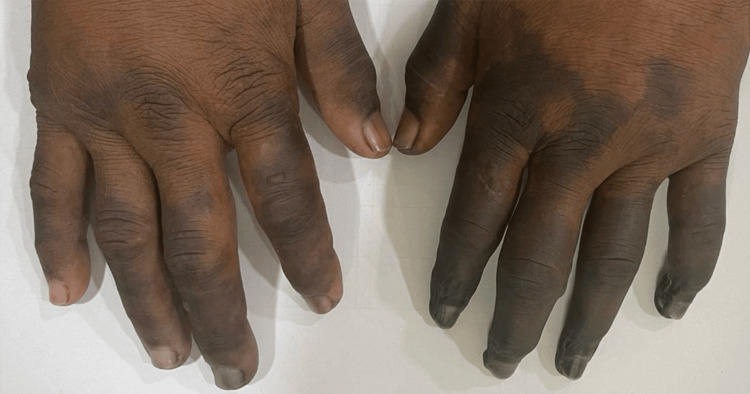
Multiple Digits Showing Ischemic Discoloration With Left 2nd to 5th Terminal Phalanx Showing Necrotic Changes

A 26-year-old female was referred from dermatology with a history of blackish discoloration of multiple digits in the bilateral upper limb for 2 weeks which was progressive (Figure 10). An associated history of fever, photosensitive rash, oral ulcers, or excessive hair fall was present eight months back. The patient gave a history of Raynaud's phenomenon for four years. No history of serositis, thrombotic events in the past pregnancy morbidity, or trauma. There was no discharge from the digits. On examination, there were no obvious findings apart from necrotic changes in digits in bilateral upper limbs. Investigations revealed positive ANA by IF, speckled pattern, positive anti-DsDNA antibody and direct Coombs test (Table 6). The patient was diagnosed with systemic lupus erythematosus and is currently on Tablet hydroxychloroquine 200 mg twice daily, low molecular weight heparin 40 mg subcutaneously once daily, and Tablet Nifedipine retard 10 mg twice daily. At present, the patient has no systemic features of SLE and the digital necrosis has not progressed any further.

**Table 6:** Investigations (Case 6) Hb: Hemoglobin; TLC: Total Leucocyte Count; Cr: Serum Creatinine; SGOT: Serum Glutamic Oxaloacetic Transaminase; SGPT: Serum Glutamic Pyruvic Transaminase; RBC: Red Blood Cells; ESR: Erythrocyte Sedimentation Rate; CRP: C-Reactive Protein; ANA by IF: Anti-nuclear Antibody by Immunofluorescence; 1:640 = Indicates the titre of ANA by IF; 3+ = indicates the strength of immunofluorescence; Homogeneous = indicates the pattern of immunofluorescence; ENA: Extractable Nuclear Antigen

Investigations	Observed Values	Reference Values
Hb (g/dl)	6.9	13 – 17 (male), 12 – 15 (female)
TLC (per microL)	3800	4000 – 10000
Platelets (per microL)	1.45 lac	1.5 – 4 lac
Cr (mg/dl)	1.1	0.6 – 1.2 (male), 0.5 – 1.0 (female)
SGOT (U/l)	23	17 – 59 (male), 14 – 36 (female)
SGPT (U/l)	21	< 50 (male), < 35 (female)
Globulin (g/dl)	3.1	2.5 – 3.5
Direct Coombs Test	Positive	Negative
Urine protein	1+	Nil
Urine RBC	Occasional	Nil
ESR (mm/hr)	70	1 – 10 (male), 3 – 15 (female)
CRP (mg/dl)	12.1	0 – 5
ANA By IF	1:640	Negative
	3+	
	homogenous	
ENA Profile	Anti-ds-DNA positive	Negative
Antiphospholipid Antibodies	Negative	Negative

Table 7 shows a summary of all cases mentioned above. 

**Table 7:** Summary of Case Series ANA: Anti-nuclear Antibodies; IF: Immunofluorescence; LAC: Lupus Anticoagulant; AcL = Anti Cardiolipin Antibody; CT: Computerized Tomography; MRSS = Modified Rodnan Skin Score; ENA: Extractable Nuclear Antigen; HRCT: High-Resolution Computerized Tomography; PFT: Pulmonary Function Test; ds-DNA = double-stranded Deoxy Rico Nucleic Acid; OD: Once a day; BD: Twice a day; scL-70: Scleroderma Antibody (Anti Topoisomerase Antibody); INR = International Normalized Ratio

No.	Patient Information	History	Examination	Investigation	Diagnosis	Treatment
1	16 years old, Male	History of polyarthritis for 2 months, Digital ischemia in left great toe (1 year back)	Multiple tender and swollen joints, No ischemic changes in digits, All peripheral pulses felt	ANA by IF positive, LAC positive, AcL antibody IgM positive	Antiphospholipid Antibody Syndrome	Heparin, Warfarin (Maintaining INR between 2 to 3), Methotrexate (with Folic Acid supplementation)
2	12 year old, Female	History of ischemic changes in the 2^nd ^toe of the left foot for 2 weeks, Similar complaints in the left great toe (amputated, 3 months back)	Black discolouration of 2^nd^ toe of left foot, All Peripheral pulses felt, No discharge, No warmth or tenderness at local site	ANA by IF positive, AcL Antibody IgM positive	Antiphospholipid Antibody Syndrome	Heparin, Warfarin (Maintaining INR between 2 to 3), Hydroxychloroquine 200 mg OD
3	26 year old, Female	History of ischemic changes in left lower limb for 1 month, Below knee amputation was done 7 days back, Acute polyarthritis for 2 days (postoperatively)	Multiple tender and swollen joints (large and small), Loss of Dorsalesis pedis pulsation on the right side, Feeble radial and brachial artery pulsation on left side	CT angiography of right lower limb showed no blood flow below the knee, Blood flow restored after balloon angioplasty	Takayasu Arteritis	Pulse steroid methylprednisolone 500 mg intravenous for 3 days, Oral prednisolone 1 mg/kg in tapering dose, Methotrexate (with Folic Acid supplementation)
4	30 year old, Female	History of swelling in both hands for 2 months, Bilaterally symmetrical inflammatory polyarthritis, Raynauds phenomenon for 3 years, ischemic discolouration of right third toe (healed, for 2 years)	Multiple tender and swollen joints, Skin tightening (MRSS score of 19), Restricted mouth opening, Digital pits	Nail fold capillaroscopy showed Giant Capillaries, ANA by IF positive, ENA profile positive for ScL -70 Antibody	Diffuse Scleroderma	Vasodilators (Calcium channel blockers), Antiplatelet drugs, Prokinetics
5	40 year old, Female	History of dry cough and breathlessness for 2 years, Multiple joint pains with morning stiffness for 6 months, Raynauds phenomenon for 6 months, Black discolouration of a left middle fingertip for 2 weeks	Multiple tender and swollen joints, Fine crepitations at base of lungs, Darkening of the skin of the anterior chest and back	ANA by IF positive, ENA profile positive for AntiCentromere antibody, HRCT showed changes of Early Interstitial lung disease, PFT showed mild restrictive changes, 2D Echo showed mild Pulmonary artery hypertension	Limited Scleroderma	Vasodilators (Calcium channel blockers), Antiplatelet drugs, Tadalafil 10 mg OD, Mycophenolate Mofetil 500 mg BD
6	26 year old, Female	History of blackish discoloration of multiple fingers for 2 weeks, Raynauds Phenomenon for 4 years	Multiple fingertips showed ischemic discoloration, Gangrenous changes in few distal phalanges	ANA by IF positive, ENA profile positive for ds-DNA antibody, Coombs test positive	Systemic Lupus Erythematosus	Hydroxychloroquine, Heparin, Vasodilators (Calcium Channel Blockers)

## Discussion

Critical digital ischemia is the consequence of a disrupted vascular pathology which may start as Raynaud's phenomenon quickly progresses to complete digital necrosis [14]. Different pathophysiological processes may end up causing digital ischemia, from thrombosis, vasculitis, and trauma to vasospasm. Every pathophysiological process mentioned requires a different therapeutic approach although the result of all these pathologies is the same. Hence, it becomes imperative to recognize digital ischemia and investigate accordingly to prevent irreversible damage to the concerned digit [1]. SLE and APLA both are complex multisystem disorders with a vast array of presentations ranging from cutaneous features to visceral manifestations such as nephritis and vasculitis. Although digital ischemia is among the less common features, 1.3% of individuals may be affected by it at any point. The mechanism behind digital ischemia in lupus and/or APLA can be due to chronic inflammation leading to atherosclerosis, vasospasm, inflammation of the vessels, or thromboembolic phenomenon. The presence of antiphospholipid antibodies is higher among people with thromboembolic events ultimately leading to digital gangrene. Data showed out of 85 cases of digital gangrene in lupus, 79 were in adults and six in children. Females were more likely to be affected by the critical digital ischemia [15]. In a study, a seven-year-old child with Lupus presented with digital ischemia and ulcers. Among other symptoms of lupus, he had skin manifestations like urticaria and fever and was positive for ANA, anti-RNP antibody, anti-Sm antibody, and anti-SSA antibody. However, no details were provided regarding Antiphospholipid antibody status in this article [11]. A study by Sonkar et al. showed a 40-year-old female with rapid onset digital ischemia in bilateral extremities with no prior history of any other lupus manifestations. On investigations, she was positive for ANA and anti-dsDNA antibodies. Tests for Antiphospholipid antibodies came out to be negative. This highlights that digital ischemia may be the presenting feature of Lupus, even in the absence of antiphospholipid antibodies [8]. A study by Nkeck et al. showed a middle-aged female with Lupus for seven years having Raynaud’s after exposure to cold. She had no other risk factors and was negative for antiphospholipid antibodies. This patient was not on any treatment for seven years with stable disease and yet suddenly developed digital ischemia [16]. In our case series, we presented two pediatric cases of antiphospholipid antibody syndrome and one case of adult lupus, all presenting with critical digital ischemia. One was a male child of 16 years of age and the other two were females. 

Takayasu vasculitis affects large vessels, mainly the pulmonary vasculature and Aorta. Usually occurs more in females and before the age of 50 years [17]. A study reported a 37-year-old female with complaints of ischemic symptoms in her left foot which was progressive and associated with neurological symptoms like numbness. She also had pain and tenderness in the affected limb with a mild fever. No other history suggestive of connective tissue disease was present. Doppler of the lower limb showed no flow in the affected foot. A blood pressure examination revealed a disparity between the two arms and a CT angiogram confirmed the narrowing of the relevant vessels thereby confirming the diagnosis of Takayasu arteritis and the patient was treated accordingly [17]. Another article by Misra et al. reported cases of Takayasu arteritis who presented with lower limb gangrene and four of the patients were amputated [18]. Roy et al. reported a 26-year-old female with ischemic changes in the right upper limb extremities with claudication. Angiography showed stenosis in the vessels (right common carotid and left subclavian) [13]. In our case series, we showed a female who was initially treated for inflammatory polyarthritis as a patient of rheumatoid arthritis, but later after her ischemic event in her left lower limb, she was re-evaluated and found to have Takayasu arteritis. This shows that detailed evaluation of patients who present with arthritis can provide vital information behind the etiology and thereby guide toward proper diagnosis. 

Digital ischemia in systemic sclerosis is by far more prominent than any other connective tissue disease and imparts a therapeutic challenge as it may be recurrent and resistant to treatment. Raynaud is the predominant cause and usually exacerbates during cold weather in 90% of individuals. The pathophysiology has two bases, one is the proliferation of the intimal layer of the vessels, and another is the presence of a thrombus in the lumen. The digital ulcers are often liable to infections and if progressive, may end up in gangrene and subsequent amputation of the digit. Certain risk factors that may exacerbate digital ulcers can be male gender, a diffuse subtype of the disease, smoking et cetera [12]. In our series, there were two cases of systemic sclerosis, one presented with arthritis and the other with respiratory symptoms. Both were later referred to Rheumatology and eventually diagnosed. Therefore, suspecting an autoimmune etiology in every case of digital ischemia is the first step toward quick diagnosis which saves the affected digit/limb in these patients. Another important point is to understand that digital ischemia represents the tip of the iceberg, and underlying disease needs to be sought in every case of digital ischemia as these patients may have undiagnosed organ manifestations due to their systemic illness. 

In our case series, we have shown how digital ischemia is presented in various departments from pediatrics, respiratory medicine, and interventional radiology to surgery. Hence, every specialty in a hospital will eventually come across a case of digital ischemia which may have been looked at as an isolated entity only to see that the underlying systemic illness sprouts later with other manifestations. This case series highlights the importance of rooting for the cause of digital ischemia till you find it as an episode of this event may not be the first and last one.

## Conclusions

Digital ischemia is a pan-specialty problem with the etiologies spreading across a vast spectrum of rheumatological disorders, many of which may present to different specialties initially, later discovered to be part of the systemic manifestation of autoimmune diseases. Hence, it becomes imperative to have a rheumatological perspective in all such cases of digital ischemia which all specialties should be aware of, and timely referral may prevent permanent loss of the digits and in some cases the entire limb. 
